# Diversity and Evolution of *Entomocorticium* (Russulales, Peniophoraceae), a Genus of Bark Beetle Mutualists Derived from Free-Living, Wood Rotting *Peniophora*

**DOI:** 10.3390/jof7121043

**Published:** 2021-12-06

**Authors:** João P. M. Araújo, You Li, Diana Six, Mario Rajchenberg, Matthew E. Smith, Andrew J. Johnson, Kier D. Klepzig, Pedro W. Crous, Caio A. Leal-Dutra, James Skelton, Sawyer N. Adams, Jiri Hulcr

**Affiliations:** 1 School of Forest, Fisheries and Geomatics Sciences, University of Florida, Gainesville, FL 32611, USA; yourreason88@gmail.com (Y.L.); ajj@ufl.edu (A.J.J.); sawyer.adams@ufl.ed (S.N.A.); hulcr@ufl.edu (J.H.); 2 Institute of Systematic Botany, The New York Botanical Garden, Bronx, NY 10458, USA; 3 Department of Ecosystem and Conservation Sciences, The University of Montana, Missoula, MT 59812, USA; diana.six@mso.umt.edu; 4 National Scientific and Technical Research Council (CONICET), Buenos Aires C1425FQB, Argentina; mrajchenberg@ciefap.org.ar; 5 Patagonian Andes Forest Research and Extension Centre (CIEFAP), Esquel 9200, Argentina; 6 Department of Plant Pathology, University of Florida, Gainesville, FL 32611, USA; trufflesmith@ufl.edu; 7 Joseph W. Jones Ecological Research Center, Odum School of Ecology, University of Georgia, Newton, GA 30602, USA; kier.klepzig@jonesctr.org; 8 Westerdijk Fungal Biodiversity Institute, Uppsalalaan 8, 3584CT Utrecht, The Netherlands; p.crous@wi.knaw.nl; 9 Section for Ecology and Evolution, Department of Biology, University of Copenhagen, 2100 Copenhagen, Denmark; caioboss@gmail.com; 10 Biology Department, The College of William and Mary, Williamsburg, VA 23185, USA; Skelto3@gmail.com

**Keywords:** wood-decaying fungi, *Russulales*, *Peniophoraceae*, mutualism, new species

## Abstract

Symbiosis between insects and fungi arose multiple times during the evolution of both groups, and some of the most biologically diverse and economically important are mutualisms in which the insects cultivate and feed on fungi. Among these are bark beetles, whose ascomycetous cultivars are better known and studied than their frequently-overlooked and poorly understood basidiomycetous partners. In this study, we propose five new species of *Entomocorticium*, fungal mutualists in the Russulales (*Basidiomycota*) that are mutualistic symbionts of scolytine beetles. We have isolated these fungi from the beetle mycangia, which are structures adapted for the selective storage and transportation of fungal mutualists. Herein, we present the most complete phylogeny of the closely related genera *Entomocorticium* and *Peniophora* and provide insights into how an insect-associated taxon (*Entomocorticium*) evolved from within a wood-decaying, wind-dispersed lineage (*Peniophora*). Our results indicate that following a transition from angiosperms to gymnosperms, fungal domestication by beetles facilitated the evolution and diversification of *Entomocorticium*. We additionally propose four new species: *Entomocorticium fibulatum* Araújo, Li & Hulcr, sp. nov.; *E. belizense* Araújo, Li & Hulcr, sp. nov.; *E. perryae* Araújo, Li & Hulcr, sp. nov.; and *E. macrovesiculatum* Araújo, Li, Six & Hulcr, sp. nov. Our findings highlight the fact that insect-fungi associations remain an understudied field and that these associations harbor a large reservoir of novel fungal species.

## 1. Introduction

Several insect groups within ants, termites, wasps, and beetles have independently evolved mutualisms with a variety of fungal lineages that help them extract nutrients from wood, an otherwise intractable substrate [1,2,3]. In many of these associations, the insects are true fungus farmers, i.e., they inoculate their fungal symbionts into the substrate and cultivate them to feed their progeny, and therefore, the fungal symbionts have become domesticated crops [4,5]. Many of these insect-cultivated fungi have evolved nutrient-provisioning adaptations and have become dependent on their partner insects for dispersal [4]. Many of these insects, including beetles, have evolved specific, highly evolved organs (mycangia) to maintain and transport their symbiotic fungi [6,7,8]. Unfortunately, fungal mutualists have thus far been studied in fewer than 5% of all bark beetles [5].

The most well-known fungi found living mutualistically with Scolytinae beetles are species within *Ophiostomatales* (*Ascomycota*). However, because these are often targeted in surveys of bark beetle fungi, a dearth of knowledge exists on other potential fungal mutualists. Surprisingly, this even includes important pest species. For example, the southern pine beetle (SPB), *Dendroctonus frontalis* (Curculionidae, Scolytinae, Hylurgini) is the most economically important pest in pine plantations across the Southern USA and Central America [9]. The biology, ecology and management of this beetle have been extensively investigated [9,10,11]. The southern pine beetle has also served as a model system for understanding symbiotic interactions between beetles and fungi [12,13]. Despite these previous investigations of symbiotic associations with *D. frontalis*, the diversity and evolutionary history of its most beneficial fungal associate—an *Entomocorticium* species (Russulaceae, Russulales, Basidiomycota)—remains obscure.

The genus *Entomocorticium* is currently comprised of eight species, all associated with Scolytinae beetles [14,15]. *Entomocorticium dendroctoni*, the type species of *Entomocorticium* [14], was described based exclusively on morphological features to accommodate a cryptic fungus that was observed growing intermingled with a blue stain fungus [14]. According to the original description, the fungus produced abundant sessile basidiospores in the galleries and pupal chambers of the mountain pine bark beetle *D. ponderosae* (mountain pine beetle) in *Pinus contorta* (lodgepole pine) [14].

The nutritional symbioses with *Entomocorticium* are only known for a few beetle species thus far [15,16]. Some of these beetles use mycangia to carry their *Entomocorticium* partners from tree to tree. Within the genus *Dendroctonus* (Curculionidae, Scolytinae, Hylurgini), some of the main documented vectors of *Entomocorticium* fungi, there are two clades of beetles with independent origins of mycangia [17,18]. One clade contains *D. ponderosae* and *D. jeffreyi*, which possess maxillary mycangia (located in the maxillae, the segmented mouthparts) wherein they carry obligate *Ophiostomatales* mutualist fungi [19]. These beetles appear to have loose associations with multiple *Entomocorticum* species that have not previously been found to be transported in mycangia. They are occasionally found in the beetle’s pupal chambers but how the fungi are disseminated is unknown [20]. The second clade of beetles, which includes *D. frontalis, D. brevicomis,* and several other species, all possess prothoracic mycangia (a tube in the inner wall of the pronotum) [21,22]. Some of these bark beetles carry *Entomocorticium* in their mycangia and for at least two, *D. frontalis* and *D. brevicomis*, the fungi are obligate nutritional mutualists. However, the fungal symbionts are unknown for many of these beetles.

Two other genera of pine beetles, only distantly related to *Dendroctonus*, are also known to be associated with *Entomocorticium*. A twig beetle, *Pityoborus comatus* (Curculionidae, Scolytinae, Corthylini), carries an *Entomocorticium* sp. in large, pubescent impressions on the sides of its prothorax that function as a mycangium [23]. *Ips avulsus* (Curculionidae, Scolytinae, Ipini) is also commonly found with an *Entomocorticium* species but how it is disseminated is not known and the presence of mycangia or other spore carrying-structures have not been investigated for this beetle [24].

Remarkably, the diversity of this important group of fungi remained uncharacterized until the early 2000s. Hsiau & Harrington (2003) were the first to show that *Entomocorticium* was a diverse fungal lineage associated solely with a group of phloem-inhabiting bark beetles that feed heavily on fungi. In addition to *E. dendroctoni,* they identified nine putative species based on their mt-SSU, ITS and IGS-1 analyses. Hsiau & Harrington [16] also suggested that the *Entomocorticium* clade was relatively young, likely having been recently derived from *Peniophora*, a genus of resupinate wood decay fungi that colonize several plant families and that rely exclusively on the wind to disperse their spores. A more recent study described seven of Hsiau & Harrington’s nine putative species of *Entomocorticium* based on morphology and molecular data from the ITS and 28S rDNA [15]. Unfortunately, no studies to date have addressed the broader evolutionary picture regarding the ecological relationships between the genera *Peniophora* and *Entomocorticium* as well as the context in which their associations with the beetle vectors and host trees might have occurred.

In this study, we propose five new species belonging to the genus *Entomocorticium* and explore the diversity and evolutionary relationships of this fungal lineage with their beetle vectors and tree hosts. In order to investigate possible evolutionary scenarios, we have built a comprehensive phylogeny based on all available data from the genera *Peniophora* (54 species) and *Entomocorticium* (13 named species, including those proposed herein) and three putative species. We tested whether *Entomocorticium* is a distinct, monophyletic genus within the order *Russulales* and what factors promoted its differentiation from the genus *Peniophora*. In terms of ecology and evolution, we investigated the beetle host spectrum across the *Entomocorticium* phylogeny and provide a hypothesis on how the association between gymnosperms, angiosperms and beetles influenced the rise of these fungal mutualists.

## 2. Material and Methods

### 2.1. Fungus Isolation

The fungi used in this study were isolated from pronotal mycangia of adult bark beetles *Dendroctonus brevicomis*, *D. frontalis*, and *Pityoborus comatus*, in the USA (California, Colorado, Florida, Louisiana, Montana, Michigan, New Mexico, South Carolina, Texas and Utah) (see [25]) and Belize (Table 1). Isolates of *Entomocorticium fibulatum* and *E. belizense* were conducted for this study, while the isolation of *E. perryae* and *E. macrovesiculatum* was previously conducted by Bracewell and Six [25]. Beetles were identified using external morphology with identification keys and images [26,27,28]. Whole beetles were surface-washed by vortexing for 1 min in 1 mL of sterile distilled water with one small drop of Tween detergent. Pronota of adult beetles were removed and crushed in a 500 µL of sterile phosphate buffer saline and vortexed for 30 s. The resulting solutions were diluted to 1:100 and 1:1000 concentrations, and each dilution was used to inoculate potato dextrose agar (PDA; Becton, Dickinson and Company, MD, USA) plates. Fungi were allowed to grow at 25 °C for 5–10 d. Representative isolates of different fungal morphotypes were placed onto new 2% potato dextrose agar (PDA) plates to obtain pure cultures and these were retained for molecular identification. In addition, we attempted to induce the production of the sexual stage by plating the isolates in Malt agar and also inoculating them in pinewood chips, but these efforts failed to promote the production of the sexual stage in all our isolates. Axenic cultures of the fungi are deposited in the culture collection (CMW) of the Forestry and Agricultural Biotechnology Institute (FABI), University of Pretoria, South Africa and in the Westerdijk Fungal Biodiversity Institute collections (CBS). Beetle remains of specimens collected in Belize or Florida were vouchered the UF Forest Entomology (UFFE) cryo-collection.

### 2.2. Morphological Observations

To access the micro-morphological features, we collected small samples of each isolate in 3–5 parts across the plate, i.e., edge, intermediate portion and center. These fungal pieces were mounted in 4% lactic acid or lacto-fuchsin and observed under an optical microscope (Zeiss Axioscope 5). Measurement of taxonomically relevant structures, e.g., vesicles and chlamydospores, were performed using the Zen software (Zeiss, Jena, Germany). The semi-permanent slides were sealed with nail polished by direct application of at least 3 layers around the cover slip edges and stored in a slide box for further observation.

### 2.3. Taxa Sampling and Sources

In order to test the relationship of *Entomocorticium* species with other genera within the order *Russulales*, we built a comprehensive phylogeny based on LSU and ITS sequences from [29,30] (Supplementary Figure S1 and Table S1). Once we established the relationship between *Entomocorticium* and *Peniophora*, we performed a second analysis including five loci, (SSU, LSU, *TEF*, ITS and IGS) consisting of 129 taxa from *Peniophora* and *Entomocorticium* species and four outgroup taxa (*Dichostereum* spp.). Sequences in the analysis included those from our isolates as well as *Peniophora* and *Entomocorticium* sequences archived in GenBank. However, the majority of taxa of our dataset (78 out of 138) were composed of only ITS and LSU rDNA due to limited data availability in GenBank for this fungal group (Table 2). As a quality control approach to confirm the identity of sequences used in this study, we subjected all sequences, including newly generated sequences of *Entomocorticium* from beetle mycangia (Table 2) to a BLAST comparison with reliable ex-types.

### 2.4. DNA Extraction, PCR Amplification, and Sequencing

Genomic DNA was extracted from fungal cultures of the new *Entomocorticium* isolates grown on PDA using the Extract-N-Amp Plant PCR kit (Sigma-Aldrich, St. Louis, MO, USA) with the modification of using 3% bovine serum albumin (BSA) as a replacement for a dilution solution. Primer combinations used for PCR amplifications were: (1) LR0R/LR5 [31] for nuclear large subunit (28S rDNA) ribosomal DNA; (2) NS1/NS4 [32] for nuclear small subunit (18S rDNA) ribosomal DNA (rDNA); (3) 983F/2218R for Translation elongation factor 1-α (TEF); (4) ITS1/ITS4 for the Internal Transcribed Spacer rDNA (ITS1-5.8S-ITS2, hereafter referred to as ITS) [33] and (5) IGS (P1/5SRNA) (Hsiau & Harrington 2003). The sequencing was performed at Eurofins. As a quality control procedure, we inspected electropherograms of each sequence individually and performed *de novo* assembling in Geneious v. 11.1.5 [34].

### 2.5. Phylogenetic Analyses

Sequence alignment was performed with MAFFT 1.4.0 [35]) separately for each marker. The alignment for each individual locus was improved manually by trimming the longer unique ends and removing gaps. The sequences were then annotated and concatenated into a single combined dataset using Geneious v. 11.1.5 [34]. Ambiguously aligned regions were excluded from phylogenetic analysis and gaps were treated as missing data. The final alignment is available in Treebase.org (http://purl.org/phylo/treebase/phylows/study/TB2:S29025). The first analysis of the order *Russulales* was composed of 145 sequences divided into four partitions: ITS1, and 28S rDNA (Supplementary Table S1). The final alignment length was 1942 bp, 683 for ITS (ITS1, 5.8S and ITS2) and 1259 bp for 28S rDNA. For the second analysis of *Peniophora* and *Entomocorticium* (Table 2), the final alignment length was 4662 bp: 1259 bp for 18S rDNA, 951 bp for 28S rDNA, 1040 bp for *TEF*, 1004 bp for ITS and 408 bp for mt-lsu. Maximum likelihood (ML) analyses were performed with RAxML v. 8.2.4 [36] on a concatenated dataset. The dataset consisted of seven data partitions, including one each for SSU, LSU, *TEF,* mt-lsu and three for ITS (ITS1, 5.8S and ITS2). The GTRGAMMA model of nucleotide substitution was employed during the generation of 1000 bootstrap replicates.

### 2.6. Ancestral Character State Reconstruction

To understand the evolutionary history of *Peniophora* and *Entomocorticium* and their associations with beetle vectors and tree hosts, we conducted ancestral character state reconstruction (ACSR) in Mesquite [37], using the best-scoring ML tree produced in RAxML. To interpret host association evolution, each taxon was coded as associated with either angiosperms or gymnosperms (*Pinaceae*). Additionally, in order to understand the evolution of the association with beetle vectors, we performed a second analysis of the association between *Entomocorticium* and six vector categories: *Dendroctonus brevicomis*, *D. frontalis, D. ponderosae, Pityoborus comatus* and *Ips avulsus*. We used maximum likelihood model MK1, as implemented in Mesquite v. 3.61 [37]. Only nodes presenting > 50% probability were displayed and used to color-code the branches on the figures.

### 2.7. Post-Analyses Graphical Display

Following the phylogenetic and ancestral character state reconstruction analyses, we used tools available in Geneious v. 11.1.5 [34] and Dendroscope [38] to optimize the tree layout. Further graphic treatment was performed in Adobe Illustrator and Procreate software in iPad Pro.

## 3. Results

**Figure 1:**
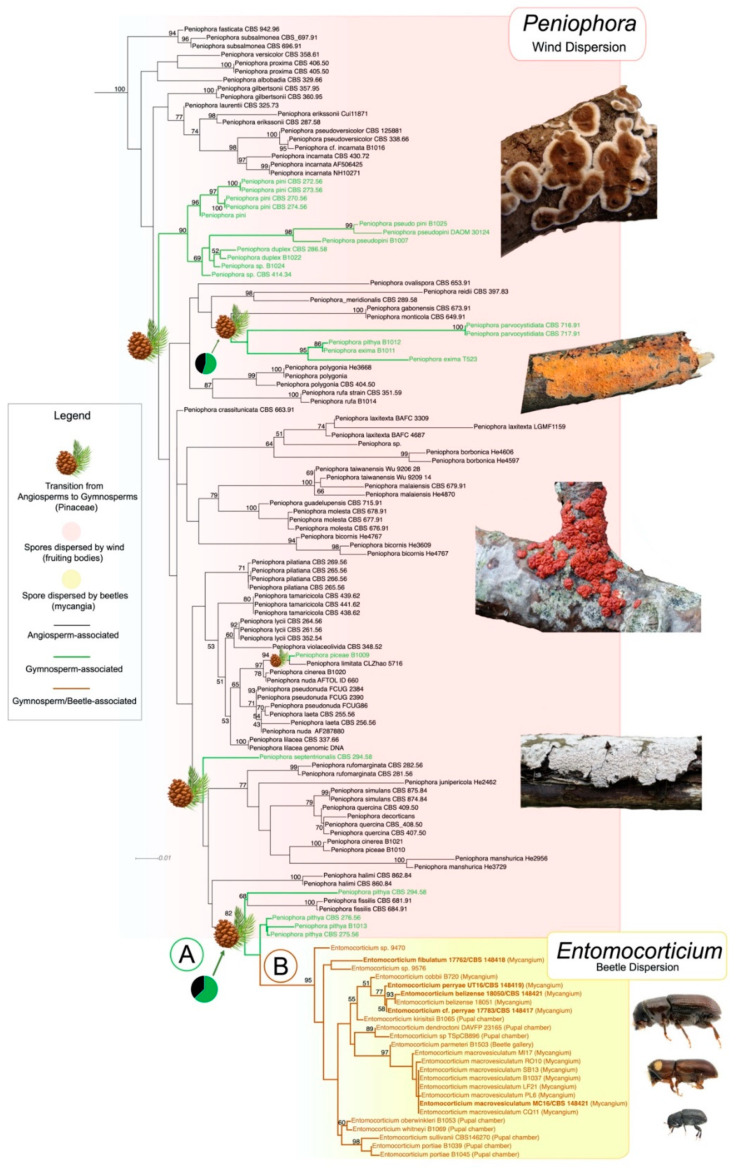
Maximum likelihood tree showing *Peniophora/Entomocorticium* clade obtained from RAxML analyses with a concatenated dataset of 5-loci (SSU, LSU, TEF, ITS and IGS). Ancestral Character State Reconstructions (ACSR) analyses based on fungal association with their plant hosts. Black branches mean association with angiosperms, green indicate an association with gymnosperms and no association with beetles, brown indicates association with gymnosperms and beetles. Pinecones indicate a transition from angiosperms to gymnosperms. Node A indicates the transition from angiosperms to gymnosperms and the origin of *Entomocorticium*, node B indicates fungal radiation following the association of *Entomocorticium* with bark beetles. Photos by Patrick Harvey, Jerzy Opioła, Eva Skific and Andrew Johnson.

To understand the species diversity and the evolutionary and ecological processes that led to the domestication of a wood-decaying fungal lineage by bark beetles, we built the most comprehensive phylogeny of the genera *Peniophora* (54 spp.) and *Entomocorticium* (17 spp.) to date. Our phylogenetic reconstruction corroborates previous studies connecting both fungal genera [15,16] (Figure 1).

**Figure 2:**
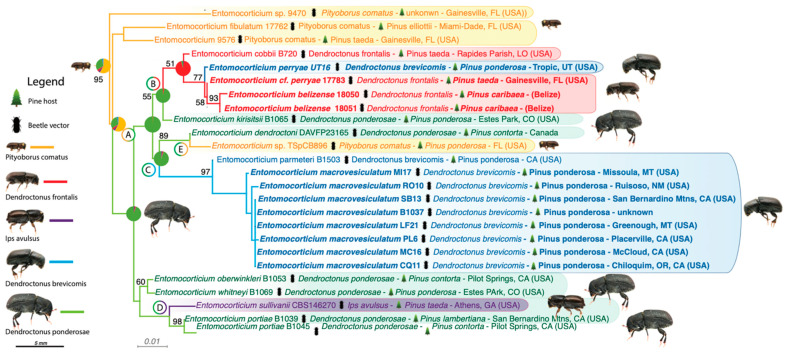
Close-up of the *Entomocorticium* clade showed in Figure 1. Character-state reconstruction of the association of *Entomocorticium* spp. with beetle vectors. Branch and boxes color mean: Yellow = *Pityoborus comatus*; Red = *Dendroctonus frontalis*; Blue = *D. brevicomis*; Green = *D. ponderosae*; Purple = *Ips avulsus*. Node A indicates transition from *Pityoborus comatus* to *Dendroctonus ponderosae*, node B from *D. ponderosa* to *D. frontalis*, node C from *D. ponderosae* to *D. brevicomis*, node D from *D. ponderosae* to *Ips avulsus* and node E from *D. ponderosae* back to *P. comatus*. Scale bar is in relation to the beetle sizes = 5 mm. Beetle photos by Andrew Johnson.

We describe an evolutionary switch from fungi with relatively complex basidiocarps that are strictly wind-dispersed (*Peniophora*) to fungi with minimal or unknown reproductive structures that are actively dispersed within beetle mycangia (*Entomocorticium*). Our ancestral character state reconstruction (ACSR) indicates that *Peniophora* is ancestrally associated with angiosperms but has transitioned to gymnosperms at least five times. Among the 54 species of *Peniophora* included in this study, only nine are associated with gymnosperms, i.e., *Peniophora duplex*, *P. exima*, *P. parvocistidiata*, *P. piceae*, *P. pini*, *P. pseudonuda*, *P. pseudo-pini*, *P. pithya* and *P. septentrionalis* (Figure 1, green branches). Our results indicate that following one of these transitions from angiosperms to gymnosperms (Figure 1, Node A), fungal domestication by bark beetles facilitated the evolution of *Entomocorticium* (Figure 1, Node B). Our data suggest that the domestication of these fungi by beetles might have promoted speciation and dissemination of this new fungal lineage across at least five beetle lineages. Currently, we have records for six beetle species associated with *Entomocorticium* (five shown in Figure 2), which might represent at least three independent origins (beetle genera) of *Entomocorticium* farming and multiple vector switches within those beetle groups.

With the current state of sampling of *Entomocorticium* we investigated the radiation of the genus with its beetle vectors. Our analysis, considering the beetle vector associations, suggests that the first beetle lineage to have domesticated an ancestor of the genus *Entomocorticium* was likely the twig beetles in *Pityoborus* (ACSR = 58%; Figure 2). After that, a transition from twig beetles to *D. ponderosae* appears to have occurred relatively soon after the initial domestication. Interestingly, *Entomocorticium* spp. found with *D. ponderosae* are not consistent, never carried in mycangia, and any association with the beetle is, therefore, most likely facultative and co-evolution is not expected. There were at least four switches after acquisition by *D. ponderosae* to other beetles, including *D. frontalis* (Figure 2 node B, ACSR = 95%) and *D. brevicomis* (Figure 2 node C, ACSR = 94%) and to other beetle genera, i.e., *Ips avulsus* (Figure 2, node D. ACSR = 88%), and a re-association with *Pityoborus* (Figure 2, node E, ACSR = 99%).

### Taxonomy

Prior to this work, the genus *Entomocorticium* was comprised of eight species: *E. dendroctoni*, *E. cobbii*, *E. kirisitsii*, *E. parmeteri*, *E. oberwinkleri*, *E. whitneyi*, *E. sullivanii* and *E. portiae* [14,15]. Distinct lineages in *Entomocorticium* can be recognized using a combination of morphology, distribution, vector-host associations and molecular markers (see Supplementary Table S2 showing inter and intraspecific genetic variation across in *Entomocorticium*). The topology of our multi-loci phylogenetic analyses revealed distinct fungal lineages associated with distinct beetle vectors and *Pinus* (Figure 2). We propose five new species of *Entomocorticium* based on all these traits combined. These new species were isolated from mycangia of *D. brevicomis*, *D. frontalis* and *Pityoborus comatus* inhabiting *Pinus ponderosa*, *P. caribaea*, *P. taeda* and *P. elliottii* in several USA states and Belize. Several additional lineages were found which are likely to be new taxa but were not described because we were unable to revive live cultures for obtaining morphology and depositing type material.

**Figure 3:**
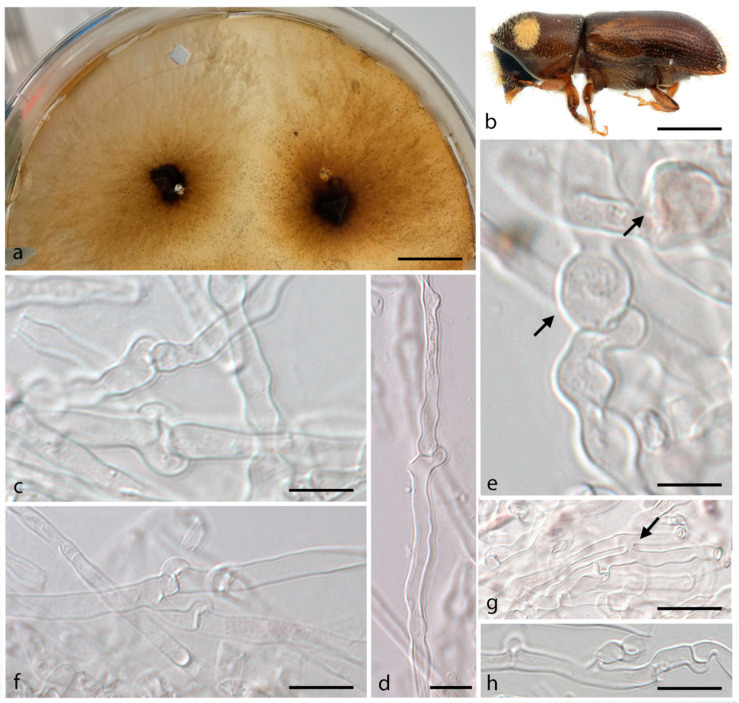
*Entomocorticium fibulatum*. (**a**) Culture aspect on PDA plate; (**b**) The beetle vector *Pityoborus comatus*; (**c**,**d**) Clamp connections; (**e**) Early stage of chlamydospores formed by a clamp connection (arrows); (**f**) Clamp connections; (**g**) Hyphae anastomosing (arrow); (**h**) Hypha exhibiting regular clamp connections. Scale bars: (**a**) = 2 cm; (**b**) = 0.5 cm; (**c**,**d**) = 4 µm; (**e**–**h**) = 5 µm.

***Entomocorticium fibulatum*** J.P.M. Araújo, Y. Li & J. Hulcr, *sp. nov.*–MycoBank MB 839833; Figure 3.

*Etymology*. The species epithet is derived from *fibula* (L. adj. f., with clamp) and refers to the abundant presence of clamp connections throughout the mycelium.

*Typus.* USA, Miami-Dade-FL, from *Pityoborus comatus* mycangium, 15 July 2015, J. Skelton, Y. Li & J. Hulcr (holotype FLAS-F-68307 (dried culture), ex-type CBS 148418 (live culture)).

*Diagnosis*. Fungus associated within *Pityoborus comatus* mycangium, inhabiting *Pinus elliottii.* Sterile hyphae exhibit abundant clamp connections throughout the mycelium.

*Sexual morph* not observed. *Asexual morph* is composed of sterile mycelium, simple or sparsely branched hyphae that are 2.1–5.8 µm wide, septate, with anastomosing hyphae and abundant clamp connections. *Hyphae* cylindrical, hyaline, sub-hyaline, forming thin-walled chlamydospore structures averaging 8 × 6 µm. Aleurioconidia not observed. Mycelial mat in culture regular, circular, pale brown becoming darker brown with age, slightly fimbriate, velvety, growing within and on the media.

*Vector*–*Pityoborus comatus* (Coleoptera, Curculionidae), Voucher UFFE: 28951.

*Host*–*Pinus elliottii* (Pinales, Pinaceae)

*Distribution*–Only recorded from Miami-Dade, FL (USA).

**Figure 4:**
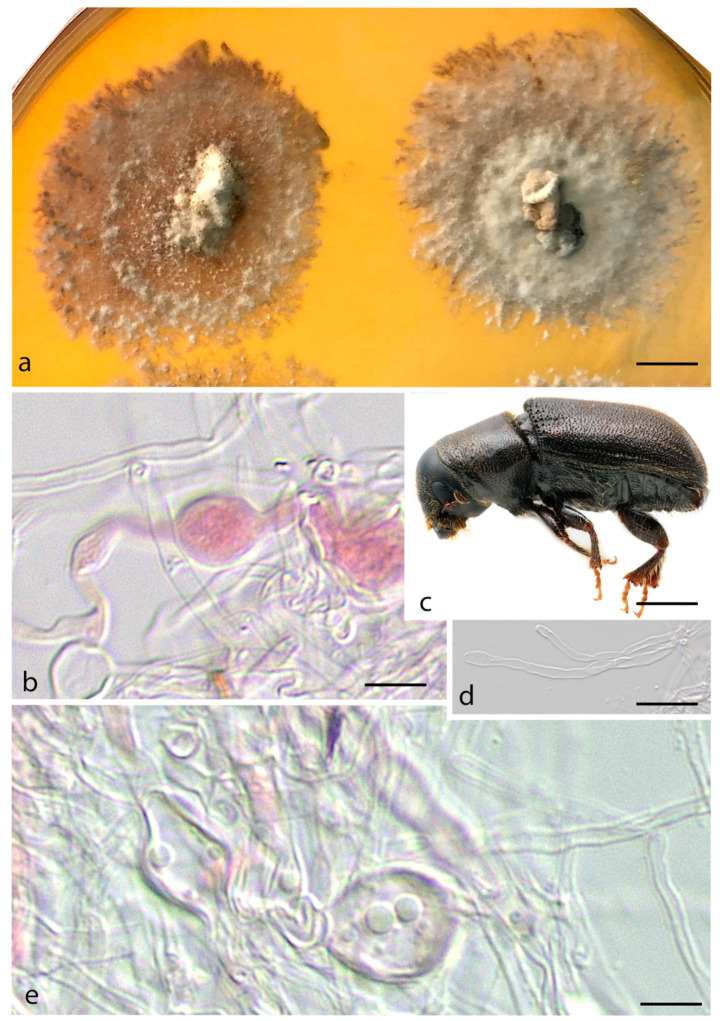
*Entomocorticium perryae.* (**a**) Culture aspect on PDA plate; (**b**) Chlamydospore in formation; (**c**) *Dendroctonus brevicomis* (vector); (**d**) Apical hyphae; (**e**) Chlamydospore. Scale bars: (**a**) = 1 cm; (**b**) = 5 µm; (**c**) = 2 mm; (**d**,**e**) = 5 µm.

***Entomocorticium perryae*** Araújo, Li, Six & Hulcr, sp. nov.–MycoBank MB 839834; Figure 4.

*Etymology.* Named after Thelma Perry, a pioneering African American female mycology technician responsible for the first description of mycangia in *Dendroctonus frontalis* and the first to report a basidiomycete from a scolytine mycangium.

*Typus.* USA, Tropic-UT, from *Dendroctonus brevicomis* mycangium, 5 July 2015, D. Six (holotype FLAS-F-68308, ex-type CBS 148419).

*Diagnosis*. The fungus associated with *Dendroctonus brevicomis* inhabiting *Pinus ponderosa*. Chlamydospores av. 6–11 × 8–13 µm.

*Sexual morph* not observed. *Asexual morph* is composed of sterile, simple, or sparsely branched hyphae that are 1.5–5 µm wide and regular or irregularly septate, clamp connections rare. *Hyphae* cylindrical and uniform, forming thin-walled chlamydospores of 6–11 × 8.2–13.5 µm. *Aleurioconidia* absent. *Cultures* floccose to dense and felty, circular, white becoming light grey to brown with age, fimbriate margin, growing within and on the media.

Vector–Dendroctonus brevicomis (Coleoptera, Curculionidae)

*Host–Pinus ponderosa* (Pinales, Pinaceae)

*Distribution*–Only recorded from Tropic, UT (USA).

Additional specimen examined: USA, Gainesville-FL, from *Dendroctonus frontalis* mycangium, 15 July 2019, J. Skelton, (FLAS-F-68306, CBS 148417 (live culture)) (as E. cf. perryae 17783): Fungus associated within *Dendroctonus frontalis* mycangium, inhabiting *Pinus taeda*. Sterile hyphae exhibit swollen hyphae, morphologically resembling those of ambrosial fungi by its clavate to globose cells that are usually irregular in size. *Asexual morph* composed of sterile, simple or branched, irregularly swollen, irregularly swollen hyphae, av. 2–5 µm width, regularly septate, clamp connections present but rare, chlamydospores absent. *Aleurioconidia* absent. Mycelial mat homogeneous, circular, light brown becoming darker with age, effuse, aerial hyphae scarce, with hyphae growing within the media.

*Vector. Dendroctonus frontalis* (Coleoptera, Curculionidae), Voucher UFFE:29184.

*Host. Pinus taeda* (Pinales, Pinaceae).

*Distribution.* Only recorded from Gainesville, FL (USA).

Note: Although we suspect that *Entomocorticium* cf. *perryae* (17783–Figure 2) is a distinct species, based on the host and vector association, we decided to take a conservative approach and include it within *E. perryae* in this study due to the very high genetic similarity (see Supplementary Table S2) and lack of morphological features. Future studies including more *E. perryae* specimens will elucidate this question.

**Figure 5:**
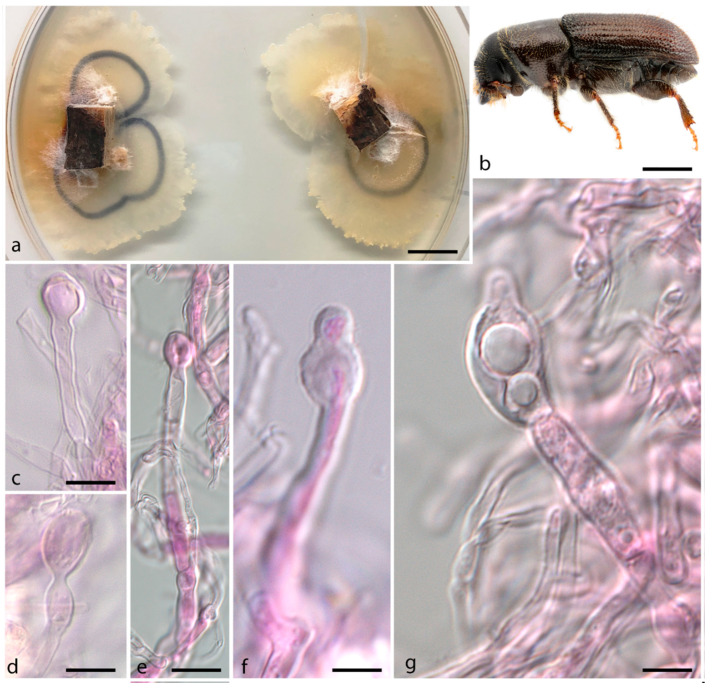
*Entomocorticium belizense*. (**a**) Culture aspect on PDA plate; (**b**) *Dendroctonus frontalis*; (**c**–**f**) Early stages of aleurioconidia; (**g**) Fully developed aleurioconidia. Scale bars: (**a**) = 1 cm; (**b**) = 0.5 cm; (**c**–**g**) = 5 µm.

***Entomocorticium belizense*** Araújo, Li & Hulcr, sp. nov.–MycoBank MB 839835; Figure 5.

*Etymology*. Named after the place of origin where it was collected, Belize.

*Typus.* Belize, Mountain Pine Ridge, from *Dendroctonus frontalis* mycangium, 21 January 2019, J. Skelton, Y. Li & J. Hulcr (holotype FLAS-F-68309 (dried culture), ex-type CBS 148420 (live culture)).

*Diagnosis.* The fungus associated within *Dendroctonus frontalis* mycangium, inhabiting *Pinus caribaea*, exhibits characteristic papillate aleurioconidia.

*Sexual morph* not observed. *Asexual morph* composed of simple or sparsely branched hyphae that are 1.5–4 µm wide and irregularly septate, clamp connections not observed. *Hyphae* cylindrical and uniform, sparsely forming thin-walled chlamydospores av. 12 × 5 µm. *Aleurioconidia* is produced at the tips of some hyphae, thick-walled, spherical to ovoid, commonly papillate, 6.5–9 × 8–17 µm. *Cultures* irregular, light cream to tan, center cottony with scarce hyphae and adpressed edges.

*Vector. Dendroctonus frontalis* (Coleoptera, Curculionidae). Voucher UFFE:30866, GenBank accession number: OL631193.

*Host. Pinus caribaea* (Pinales, Pinaceae).

*Distribution.* Only recorded from Belize.

Additional specimens examined: Belize, Mountain Pine Ridge, from *Dendroctonus frontalis* (Voucher UFFE:30867) mycangium, 21 January 2019, J. Skelton, Y. Li & J. Hulcr (18051).

**Figure 6:**
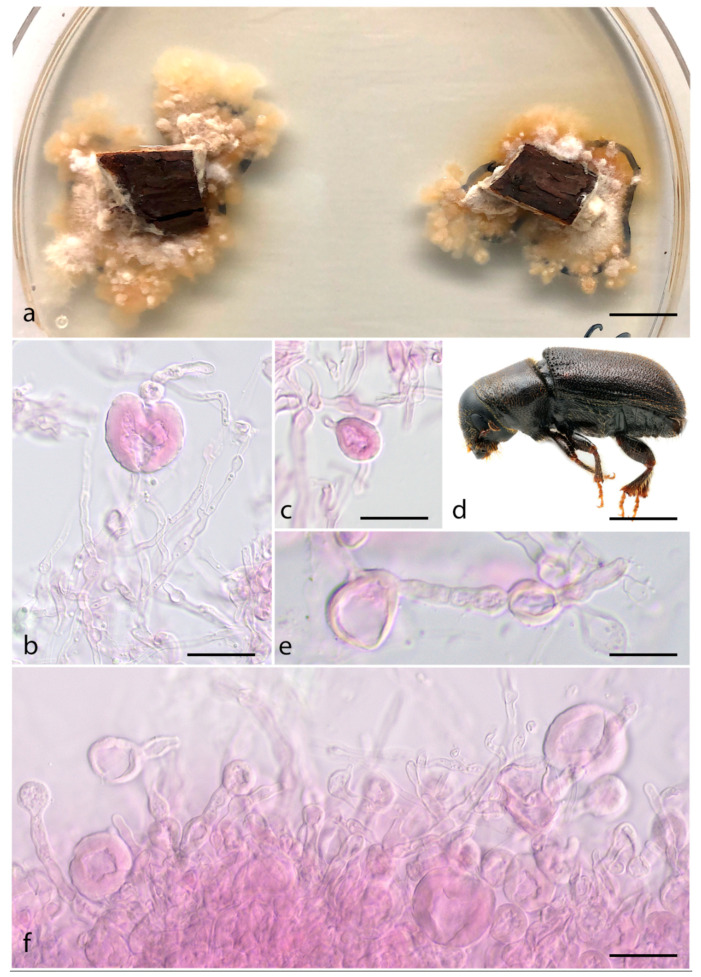
*Entomocorticium macrovesiculatum*. (**a**) Culture aspect, including two pieces of pine wood. Black line on the underside of the Petri dish indicates culture diameter on 22 December 2020, photo taken on 21 January 2021; (**b**,**c**) Chlamydospores; (**d**) *Dendroctonus brevicomis*, the vector; (**e**,**f**) Vegetative hyphae and chlamydospore-like/vesicles. Scale bars: (**a**) = 1 cm; (**b**) = 30 µm; (**c**) = 20 µm; (**d**) = 2 mm; (**e**) = 15 µm; (**f**) = 30 µm.

***Entomocorticium macrovesiculatum*** Araújo, Li, Six & Hulcr, sp. nov.–MycoBank MB 839837; Figure 6.

*Etymology.* The name refers to the large vesicles commonly seen in this species.

*Typus.* USA, McCloud-CA, from *Dendroctonus brevicomis* mycangium, July 2014, D. Six & R. Bracewell (holotype FLAS-F-68310 (dried culture), ex-type CBS 148421 (live culture)).

*Diagnosis.* The fungus associated within *Dendroctonus brevicomis* mycangium, inhabiting *Pinus ponderosa*, exhibiting abundant large vesicles.

*Sexual morph* not observed. *Asexual morph* is composed of branched hyphae that are 2–6 µm wide and regularly septate, clamp connections present but rare. *Hyphae* cylindrical, often swollen, monilioid, sparsely forming abundant thin-walled vesicles 13 × 37 µm, commonly bursting when mounted for light microscopy. *Aleurioconidia* terminal or intercalary within hyphae, apparently produced by the enlargement of single cells, capitate to ovoid, abundant, 5.5–11 × 7–15 µm. *Cultures* irregular, white to light cream to tan, cottony center with lacunose and viscous margins.

Vector. Dendroctonus brevicomis (Coleoptera, Curculionidae)

*Host. Pinus ponderosa* (Pinales, Pinaceae)

*Distribution.* Recorded from several sites across the Western USA: Chiloquim (OR), Greenough (MT), McCloud (CA), Missoula (MT), Placerville (CA), Ruisoso (NM) and San Bernardino Mountains (CA).

Additional specimens examined: USA, Missoula-MT, from *Dendroctonus brevicomis* mycangium, 17 January 2019, D. Six & R. Bracewell (MI17); USA, Placerville-CA, from *Dendroctonus brevicomis* mycangium, 20 February 2019, D. Six & R. Bracewell (PL6). USA, Ruisoso-NM, from *Dendroctonus brevicomis* mycangium, 10 January 2019, D. Six & R. Bracewell (RO10).

## 4. Discussion

In order to understand the evolution of symbiotic relationships, it is important to consider what factors have been involved in the acquisition of new hosts and vectors [39]. Host shifts by microbial symbionts are often associated with species diversification driven by the exploitation of new adaptive zones [40]. In the case of *Entomocorticium* and bark beetles, our results indicate a considerable diversity of fungal lineages within *Entomocorticium* with each species consistently associated with a particular taxon of bark beetles and their host pines.

Our phylogenetic results agree with the previously published phylogeny of *Entomocorticium* [15,16]. However, our study aimed to be more inclusive and provide further clarification regarding the evolutionary pathways that might have facilitated the origin of the genus *Entomocorticium* and promoted its further speciation. We propose a hypothesis of an evolutionary transition from a strictly wood-decaying, wind-dispersed fungal lineage (*Peniophora*) to a beetle-associated lineage engaged in highly selective vertical transmission through mycangia (*Entomocorticium*). We also provide new hypotheses on how beetle species involved in these symbiotic relationships likely played a crucial role in promoting diversity within this fungal group.

Our findings support *Entomocorticium* as a monophyletic fungal lineage that exhibits common morphological, molecular and ecological traits. Therefore, we are convinced that *Entomocorticium* should be treated as a separate genus from *Peniophora*, although that renders *Peniophora* a polyphyletic group. We hope that this study encourages further efforts to elucidate the relationships within *Peniophora*, which would ultimately result in a new taxonomic arrangement for the genus.

### 4.1. How Did Such Relationships Arise?

Our results indicate that most species within *Peniophora*, the genus from which *Entomocorticium* is derived, are broadly associated with angiosperms with at least five transitions to gymnosperms, particularly *Pinus* (Figure 1). Following one of these transitions (Figure 1 node A), the ancestor of *Entomocorticium* (related to *Peniophora pithya*) encountered bark beetles and transitioned to dissemination via beetle vectors. Given that *Peniophora* is a group of wood-rotting fungi that colonize and degrade dead wood, initial encounters between a member(s) of this group and bark beetles likely occurred in recently killed or moribund tree tissues. While *Entomocorticum* is likely undersampled in our analysis, our results indicate that twig beetles that exploit moribund phloem on shaded-out pine twigs (e.g., *Pityoborus*) were among the earliest vectors of these fungi (Figure 2).

The subsequent switches to new beetle vectors were likely facilitated by co-colonization of pine phloem by multiple species of bark beetles, resulting in exposure of the fungus to a diverse vector pool. Co-colonization of trees, i.e., niche overlap, is common in bark beetles and can result in exposure to a diverse pool of potential symbionts [41]. Shifts to new hosts may have driven both symbiont and beetle diversification in at least some cases by allowing the exploitation of new adaptive zones. Host-shift events driven by niche overlap are relatively common in fungi, especially within Hypocreales [39,42,43,44,45]. In the case of *Entomocorticium* and bark beetles, our results indicate a considerable diversity of lineages of these fungi, with each species consistently associated with a particular taxon of scolytine beetles in *Pinus.*

### 4.2. Distinct Associations across Bark Beetles and Entomocorticium

Not all symbioses between *Entomocorticium* and bark beetles are the same. There is a range of dependencies varying from loose and facultative (e.g., *D. ponderosae*) to obligate (e.g., *D. frontalis* and *D. brevicomis*) [46]. Likewise, the effects of the fungi on beetle fitness are not clear. For example, several species of *Entomocorticium* have been isolated from the pupal chambers of *D. ponderosae* and these have been suggested to be nutritional mutualists [15,16]. However, these fungal species have sporadic distributions with *D. ponderosae* [47,48] and have never been isolated from their mycangia [8,49], despite numerous isolations from beetles collected in many locations. Additionally, these fungi have been only rarely isolated from the beetle’s exoskeleton, suggesting the beetle may be an inefficient vector and the beneficial aspects of this symbiosis to the beetle, if any, is unreliable.

In contrast, *D. frontalis* and *D. brevicomis* are obligately associated with *Entomocorticum* species and these fungi provide crucial nutrients for the development of beetle larvae. The association of *D. brevicomis* with *Entomocorticium* is ancient and highly coevolved with the fungi co-speciating along with the host beetle in response to a period of isolation during glaciation [49]. Vertical transmission via highly selective mycangia enforces fidelity and reduces the potential for invasion by new lineages [46,50,51,52,53,54,55].

Regarding *Pityoborus comatus* and *Ips avulsus*, both species have been studied much less than *Dendroctonus*, but observational evidence of larval development suggests that they are completely mycophagous, at least in the larval stage. Some *Ips* species appear to be dependent on *Ophiostoma* species for nutrition [53] and this may also be the case for those that associate with *Entomocorticium*. However, most aspects of this association have not been investigated, especially regarding mycangia or other structures that facilitate fungal dissemination and little is known about specificity and nutritional effects.

### 4.3. Distinct Functional Traits in Basidiomycota and Ascomycota Associated with Bark Beetles

The association of *Entomocorticium* (Basidiomycota) with conifer-colonizing bark beetles is clearly limited compared to conifer-colonizing bark beetles occurring with Ophiostomatales (Ascomycota), which are ubiquitous worldwide [15]. For many bark beetles, Ophiostomatales fungal symbionts are facultative or obligate nutritional mutualists. The necrotrophic nature of many of these fungi allows them to survive and grow in a dying tree host during the early colonization phase of a tree and then to exploit dead tree tissues over the longer period of larval and fungal mycelial development [5]. Ophiostomatales do not degrade cellulose and lignin, which limits them to foraging for amino acids and simple carbohydrates [53].

On the other hand, the Basidiomycota symbiont species, such as those in the genera *Entomocorticium* and *Peniophora*, can actively decay the structural components of wood. Both genera are saprobic and do not invade living tissues, as demonstrated for the *Entomocorticium* species associated with *D. brevicomis* [53]. While they also consume amino acids and simple carbohydrates for energy and growth, they use these resources to support the degradation of cellulose and lignin, resulting in greater access to resources within the tree. These different qualities between the Ascomycota and Basidiomycota associates of bark beetles are not trivial and are critical to understanding the development and maintenance of such novel symbioses within bark beetles as a whole. Differences in growth within trees and the ability to access and acquire nutrients indicate different pathways to exploit wood as a niche and potentially to reduce niche overlap and competition [53].

### 4.4. Domestication of Entomocorticium by Beetles Facilitated the Loss of Morphological Traits

The transition from free-living (*Peniophora*) to beetle-associated (*Entomocorticium*) coincided with a transition to moribund phloem: a resource that presents benefits, as well as costs. Tree parts, such as moribund phloem are relatively free of competition and are more nutritious than dead wood or woody debris. However, moribund phloem is still alive and chemically defended and is also spatially patchy and intermittently available. Therefore, exploitation of such a resource is greatly facilitated by association with an agile insect vector. The optimal resource for the vector and the fungus are hence similar.

The overall loss of morphological complexity from *Peniophora* to *Entomocorticium* species is consistent with the loss of morphological features in other beetle-associated fungi [54]. Likewise, a reduction in sexual reproduction is consistent with predictions for microbes involved in mutualisms [51,52,53]. *Peniophora* are corticoid fungi that reproduce sexually and exhibit a broad diversity of basidiome morphologies (e.g., resupinate, effused, membranaceous, ceraceous, etc.), colors (e.g., reddish, orange, pink, violaceous, greyish, yellow, lilac, etc.) and colonize wood of a broad variety of plant hosts [56,57]. In contrast, *Entomocorticium* are restricted to beetle-colonized *Pinus* and only form simple whitish mycelial mats, often supporting the production of large numbers of asexual spores (chlamydospores, aleurioconidia) and with sexual spores (basidiospores) formed only rarely or not at all [14,15,16]. Basidia, when they do form, have been described as lacking Buller’s drops reflecting their production inside the tree with no potential for wind dispersal. However, as with other putative asexual mutualists, evidence of rare recombination events can be found, potentially maintained to reduce the effects of Muller’s ratchet predicted for fully asexual species [58,59].

Bark beetles are tremendously important evolutionarily, ecologically, and economically, and their complex relationships with trees and fungi are beginning to be better understood [60,61]. The descriptions of new species we provide as well as their relationships are noteworthy. They expand upon recent descriptions from Harrington et al. [15], indicating greater complexity and diversity of fungal associates of *Dendroctonus* and other bark beetle species. This work also furthers understanding of the players in this group of model organisms for the study of symbiosis.

## 5. Conclusions

The genus *Entomocorticium* provides an interesting insight into the origins of insect microbial mutualisms. This lineage of Basidiomycota has arisen quite successfully from a wood-decaying ancestor (*Peniophora*) within a matrix of pre-existing symbioses between several lineages of Ascomycota fungi and their beetle vectors [5]. Targeted sampling for *Entomocorticium* across a variety of bark beetles with various tree colonization strategies, careful investigation of fungal vectoring capacity and specialized structures of beetles, and studies on the effects of the fungi on beetle fitness via nutrient provisioning should be a focus of future investigations into beetle-fungus symbioses. This is particularly true for *Entomocorticium* associated with *P. comatus*, a beetle which has not yet been found to associate with fungi in Ophiostomatales (Ascomycota), and also with *D. ponderosae*, a beetle which has not yet been shown to harbor *Entomocorticium* symbionts within the mycangia, only from its galleries. Genetic and morphological descriptions of the fungi can provide additional information on symbiosis type and strength, as well as provide a better understanding of the functional morphology of these fungal lineages and how they evolved. Furthermore, the diversity of fungi with bark beetles in *Pinus* in Mexico and Central America, which are almost completely unsampled, should be specially targeted. These regions exhibit amazing diversity of pines and bark beetles, and most likely fungal symbionts as well. For example, Mexico alone has 43 species of *Pinus* with a myriad of unknown beetle-fungus associations [62] and these diverse pine forests most likely harbor the largest reservoirs of these intriguing, fascinating and ecologically important fungi.

## Figures and Tables

**Table 1:** List of isolates obtained in this study and other *Entomocorticium* isolates included in the analyses.

Species	Voucher (Extype)	Beetle Vector	Tree Host	Isolate Origin	Material Source	Reference
*Entomocorticium belizense*	18050 (CBS 148421)	*Dendroctonus frontalis*	*Pinus caribaea*	Belize	Mycangium	This study
	18051	*Dendroctonus frontalis*	*Pinus caribaea*	Belize	Mycangium	This study
*Entomocorticium cobbii*	B720	*Dendroctonus frontalis*	*Pinus taeda*	Rapides Parish, LO, USA	Mycangium	Harrington et al. (2021)
*Entomocorticium dendroctoni*	DAVFP 23165	*Dendroctonus ponderosae*	*Pinus ponderosa*	British Columbia, Canada	Pupal Chamber	Whitney et al. (1987)
*Entomocorticium fibulatum*	17762 (CBS 148418)	*Pityoborus comatus*	*Pinus elliotii*	Miami-Dade, FL, USA	Mycangium	This study
*Entomocorticium perryae*	UT16 (CBS 148419)	*Dendroctonus brevicomis*	*Pinus ponderosa*	Tropic, UT, USA	Mycangium	This study
*Entomocorticium kirisitsii*	B1065	*Dendroctonus ponderosae*	*Pinus ponderosa*	Estes Park, CO, USA	Pupal Chamber	Harrington et al. (2021)
*Entomocorticium macrovesiculatum*	PL6	*Dendroctonus brevicomis*	*Pinus ponderosa*	Placerville, CA, USA	Mycangium	This study
	LF21	*Dendroctonus brevicomis*	*Pinus ponderosa*	Greenough, MT, USA	Mycangium	This study, Bracewell and Six (2014)
	CQ11	*Dendroctonus brevicomis*	*Pinus ponderosa*	Chiloquim, OR, USA	Mycangium	This study, Bracewell and Six (2014)
	MI17	*Dendroctonus brevicomis*	*Pinus ponderosa*	Missoula, MT, USA	Mycangium	This study, Bracewell and Six (2014)
	Ro10	*Dendroctonus brevicomis*	*Pinus ponderosa*	Ruisoso, NM, USA	Mycangium	This study, Bracewell and Six (2014)
	SB13	*Dendroctonus brevicomis*	*Pinus ponderosa*	San Bernardino Mtns, CA, USA	Mycangium	This study, Bracewell and Six (2014)
	MC16 (CBS 148421)	*Dendroctonus brevicomis*	*Pinus ponderosa*	McCloud, CA, USA	Mycangium	This study, Bracewell and Six (2014)
*Entomocorticium oberwinkleri*	B1053	*Dendroctonus ponderosae*	*Pinus contorta*	Pilot Springs, CA, USA	Pupal Chamber	Harrington et al. (2021)
*Entomocorticium parmeteri*	B1503	*Dendroctonus brevicomis*	*Pinus ponderosa*	Tuolumme County, CA, USA	Gallery	Harrington et al. (2021)
*Entomocorticium* cf. *perryae*	17783 (CBS 148417)	*Dendroctonus frontalis*	*Pinus taeda*	Gainesville, FL, USA	Mycangium	This study
*Entomocorticium portiae*	B1039	*Dendroctonus ponderosae*	*Pinus lambertiana*	Blodgett Res. Forest, CA, USA	Pupal Chamber	Harrington et al. (2021)
*Entomocorticium portiae*	B1060	*Dendroctonus ponderosae*	*Pinus contorta*	San Bernardino Mts., CA, USA	Pupal Chamber	Harrington et al. (2021)
*Entomocorticium* sp.	MMF-4485	*Pitioborus comatus*	*Pinus ponderosa*	Florida	Pupal Chamber	Harrington et al. (2021)
*Entomocorticium* sp.	9470	*Pityoborus comatus*	*unknown*	Gainesville, FL, USA	Mycangium	This study
*Entomocorticium* sp.	9576	*Pityoborus comatus*	*Pinus taeda*	Gainesville, FL, USA	Mycangium	This study
*Entomocorticium sullivanii*	B1252	*Ips avulsus*	*Pinus taeda*	Athens, GA, USA	Pupal Chamber	Harrington et al. (2021)
*Entomocorticium whitneyi*	B1069	*Dendroctonus ponderosae*	*Pinus ponderosa*	Estes Park, CO, USA	Pupal Chamber	Harrington et al. (2021)

**Table 2:** Species used in the *Russulales* analyses and their GenBank accession numbers.

Species	Host	Voucher	SSU	ITS	LSU	TEF	Citation
*Entomocorticium belizense*	*Pinus caribaea*	18050	–	MZ098132	MZ098117	–	This study
		18051	–	MZ098133	MZ098116	–	This study
*Entomocorticium cobbii*	*Pinus taeda*	B720	–	MT741707	MT741692	–	Harrington et al. (2021)
*Entomocorticium fibulatum*	*Pinus elliottii*	17762	MZ098147	MZ098135	MZ098120	–	This study
*Entomocorticium perryae*	*Pinus ponderosa*	UT16	MZ098145	MZ098123	MZ098118	MZ144591	This study, Bracewell and Six (2014)
*Entomocorticium kirisitsii*	*Pinus ponderosa*	B1065	–	MT741714	MT741699	–	Harrington et al. (2021)
*Entomocorticium macrovesiculatum*	*Pinus ponderosa*	MI17	MZ098143	MZ098129	–	MZ144589	This study, Bracewell and Six (2014)
		RO10	MZ098149	MZ098130	MZ098108	MZ144590	This study, Bracewell and Six (2014)
		SB13	MZ098141	MZ098125	MZ098110	MZ144586	This study, Bracewell and Six (2014)
		B1037	MZ098138	MZ098124	MZ098109	MZ144585	This study, Bracewell and Six (2014)
		LF21	MZ098139	–	MZ098113	MZ144587	This study, Bracewell and Six (2014)
		PL6	MZ098140	MZ098126	MZ098114	MZ144588	This study
		MC16	MZ098144	MZ098128	MZ098112	–	This study, Bracewell and Six (2014)
		CQ11	MZ098142	MZ098127	MZ098111	–	This study, Bracewell and Six (2014)
*Entomocorticium oberwinkleri*	*Pinus contorta*	B1053	–	MT741712	MT741697	–	Harrington et al. (2021)
*Entomocorticium parmeteri*	*Pinus ponderosa*	B1503	–	MT741709	MT741694	–	Harrington et al. (2021)
*Entomocorticium* cf. *perryae*	*Pinus taeda*	17783	MZ098146	MZ098131	MZ098115	MZ144592	This study
*Entomocorticium portiae*	*Pinus lambertiana*	B1039	–	MT741710	MT741695	–	Harrington et al. (2021)
	*Pinus contorta*	B1045	–	MT741711	MT741696	–	Harrington et al. (2021)
*Entomocorticium* sp.	*Pinus taeda*	9576	MZ098148	MZ098134	–	MZ144593	This study
*Entomocorticium* sp.	*Pinus ponderosa*	TSpCB896	–	AF119510	–	–	Harrington et al. (2021)
*Entomocorticium sullivanii*	*Pinus taeda*	CBS 146270	–	MT741715	MT741700	–	Harrington et al. (2021)
*Entomocorticum dendroctoni*	*Pinus contorta*	DAVFP 23165	–	AF119506	–	–	Hsiau & Harrington (2003)
*Entomocorticum whitneyi*	*Pinus ponderosa*	B1069	–	MT741713	MT741698	–	Harrington et al. (2021)
*Peniophora albobadia*	Angiosperms	CBS 329.66	–	MH858809	MH870448	–	Andreasen & Hellenberg (2009)
*Peniophora aurantiaca*	*Alnus* (*Betulaceae*)		–			–	Boidin (1994)
*Peniophora bicornis*	*Pentaclethra* (*Fabaceae*), *Musanga* (*Urticaceae*), *Anthocleista* (*Gentianaceae*), *Casuarina* (*Casuarinaceae*), *Acacia* (*Fabaceae*), Acanthophoenyx (*Areceae*)	He4767	–	MK588764	MK588804	–	Boidin et al. (1991)
		He3609	–	MK588763	MK588803	–	Boidin et al. (1991)
*Peniophora borbonica*	*Hypericum* (*Hypericaceae*), *Acacia* (*Fabaceae*), *Fuchsia* (*Onagraceae*)	He4597	–	MK588766	MK588806	–	Boidin et al. (1991)
		He4606	–	MK588765	MK588805	–	Boidin et al. (1991)
*Peniophora cinerea*	“Angiosperms and Gymnosperms”	B1020	–	MN475151	MN475818	–	Andreasen & Hellenberg (2009)
*Peniophora crassitunicata*	*Morinda* (*Rubiaceae*), *Schinus* (*Anacardiaceae*), *Casuarina* (*Casuarinaceae*), *Lantana* (*Verbenaceae*), *Tylophora* (*Apocynaceae*), *Acanthophoenyx* (*Arecaceae*), *Scaevola* (*Goodeniaceae*)	CBS 663.91	–	MH862292	MH873972	–	Boidin et al. (1991)
*Peniophora duplex*	Gymnosperm “similar to *P. pini*/*pseudo-pini*”	CBS 286.58	–	MH857787	MH869321	–	Andreasen & Hellenberg (2009)
		B1022	–	MN475153	MN475820	–	Andreasen & Hellenberg (2009)
*Peniophora eriksonii*	*Alnus glutinosa* (*Betulaceae*)	CBS 287.58	–	MH857788	MH869322	–	Boidin (1994)
		Cui11871	–	MK588771	MK588811	–	Boidin (1994)
*Peniophora exima*	*Abies* (*Pinaceae*)	B1012	–	MN475159	MN475826	–	Boidin (1994)
		B1011	–	MN475155	MN475821	–	Boidin (1994)
		T523	–	MK588772	MK588812	–	Boidin (1994)
*Peniophora fasticata*	Angiosperms	CBS 942.96	–	MH862624	–	–	Andreasen & Hellenberg (2009)
*Peniophora fissilis*	*Cryptomeria* (*Cupressaceae*), *Lantana* (*Verbenaceae*)	CBS 681.91	MZ233430	MH862298	MH873975	–	Boidin et al. (1991)
		CBS 684.91	MZ233431	MH862299	MH873976	–	Boidin et al. (1991)
*Peniophora gabonensis*	*Pandanus* (*Pandanaceae*)	CBS 673.91	–	MH862293	–	–	Andreasen & Hellenberg (2009)
*Peniophora gilbertsonii*	*Prosopis juriflora* (*Fabaceae*), *Baccharis* (*Asteraceae*), *Cercidium* (*Fabaceae*), *Condalia* (*Rhamnaceae*), *Fouquieria* (*Fouquieraceae*)	CBS 357.95	–	MH862528	MH874164	–	Boidin et al. (1991)
		CBS 360.95	–	MH862530	MH874165	–	Boidin et al. (1991)
*Peniophora guadelupensis*	Leguminosae	CBS 715.91	–	MH862304	MH873977	–	Andreasen & Hellenberg (2009)
*Peniophora halimi*	*Atriplex* (*Amaranthaceae*)	CBS 862.84	–	MH861843	MH873531	–	Andreasen & Hellenberg (2009)
		CBS 860.84	–	MH861842	MH873530	–	Andreasen & Hellenberg (2009)
*Peniophora incarnata*	On angiosperms, rarely on Gymnosperms	B1016	–	MN475156	MN475822	–	Andreasen & Hellenberg (2009)
		CBS 430.72	–	MH860518	MH872230	–	Andreasen & Hellenberg (2009)
		AF506425	–	AF506425	–	–	Andreasen & Hellenberg (2009)
		NH10271	–	AF506425	–	–	Andreasen & Hellenberg (2009)
*Peniophora junipericola*	*Juniperus*	He2462	–	MK588773	MK588813	–	Boidin (1994)
*Peniophora laeta*	*Carpinus* (*Betulaceae*), *Ostrya* (*Betulaceae*)	CBS 256.56	–	MH857617	MH869165	–	Andreasen & Hellenberg (2009)
		CBS 255.56	–	MH857616	MH869164	–	Andreasen & Hellenberg (2009)
*Peniophora laurentii*	*Populus* (*Salicaceae*), *Betula* (*Betulaceae*), *Salix* (*Salicaceae*)	CBS 325.73	–	–	MH872397	–	Boidin (1994)
*Peniophora laxitexta*	Angiosperms	BAFC 3309	–	FJ882040	–	–	Andreasen & Hellenberg (2009)
		LGMF1159	–	JX559580	–	–	Andreasen & Hellenberg (2009)
		BAFC 4687	–	MN518328	–	–	Andreasen & Hellenberg (2009)
*Peniophora lilacea*	*Celtis* (*Cannabaceae*), *Staphylea* (*Staphyleaceae*), *Alnus* (*Betulaceae*), *Gleditsia* (*Fabaceae*), *Fraxinus* (*Olaceae*)	CBS 337.66	–	MH858813	MH870452	–	Boidin (1994)
		CBS 337.66	–	MH858813	MH870452	–	Boidin (1994)
*Peniophora limitata*	*Fraximus*, *Syringa*, *Ligustrum*, *Phillyrea*	CLZhao 5716	–	MK269148	–	–	Boidin (1994)
*Peniophora lycii*	Unkonwn	CBS 264.56	–	MH857624	MH869169	–	Andreasen & Hellenberg (2009)
		CBS 261.56	–	MH857621	MH869167	–	Andreasen & Hellenberg (2009)
		CBS 352.54	–	MH857357	MH868899	–	Andreasen & Hellenberg (2009)
*Peniophora malaiensis*	*Calophyllum* (*Calophyllaceae*)	CBS 679.91	–	MH862297	MH873974	–	Andreasen & Hellenberg (2009)
		He4870	–	MK588775	MK588815	–	Andreasen & Hellenberg (2009)
*Peniophora manshurica*	*Quercus* (*Fagaceae*)	He2956	–	MK588776	MK588816	–	Andreasen and Hellenberg (2009)
		He3729	–	MK588777	MK588817	–	
*Peniophora meridionalis*	*Quercus*, *Cistus* (*Cistaceae*), *Lentiscus*, *Eucalyptus* (*Myrtaceae*), *Erica* (*Ericaceae*)	CBS 289.58	–	MH857789	MH869323	–	Boidin et al. (1991)
*Peniophora molesta*	Unknown	CBS 678.91	–	MH862296	–	–	Andreasen & Hellenberg (2009)
		CBS 677.91	–	MH862295	–	–	Andreasen & Hellenberg (2009)
		CBS 676.91	–	MH862294	MH873973	–	Andreasen & Hellenberg (2009)
*Peniophora monticola*	*Hypericum* (*Hypericaceae*), *Dombeya* (*Malvaceae*)	CBS 649.91	–	MH862289	MH873970	–	Boidin et al. (1991)
*Peniophora nuda*	Angiosperms, rarely Gymnosperms	AFTOL_ID_660	–	DQ411533	DQ435788	–	Andreasen & Hellenberg (2009)
*Peniophora ovalispora*	*Acacia* (*Acaciae*), *Cryptomeria* (*Cupressaceae*), *Fuchsia* (*Onagraceae*), *Solanum* (*Solanaceae*), *Cyathea* (Fern)	CBS 653.91	–	MH862290	MH873971	–	Boidin et al. (1991)
*Peniophora parvocystidiata*	*Pinus* (*Pinaceae*)	CBS 716.91	–	MH862305	MH873978	–	Andreasen & Hellenberg (2009)
		CBS 717.91	–	MH862306	MH873979	–	Andreasen & Hellenberg (2009)
*Peniophora piceae*	*Abies*, *Pseudotsuga* (*Pinaceae*)	B1010	–	MN475158	MN475825	–	Boidin (1994)
		B1009	–	MN475157	MN475824	–	Boidin (1994)
*Peniophora pilatiana*	*Quercus*, *Cistus*, *Nerium*, *Vitis*, *Prunus*, *Pistacia*, *Olea*, *Rhammus*, *Salix*, *Eucalyptus*, *Ilex*	CBS 269.56	–	MH857627	MH869172	–	Boidin (1994)
		CBS 265.56	–	MH857625	MH869170	–	Boidin (1994)
		CBS 266.56	–	MH857626	MH869171	–	Boidin (1994)
*Peniophora pini*	*Pinus sylvestris* (*Pinaceae*)	CBS 272.56	–	CBS 272.56	MH869175	–	Gibson (1960)
		CBS 273.56	–	MH857631	MH869176	–	Gibson (1960)
		CBS 270.56	–	MH857628	MH869173	–	Gibson (1960)
		CBS 274.56	–	MH857632	MH869177	–	Gibson (1960)
		CBS 414.34	–	MH855589	MH867099	–	Gibson (1960)
*Peniophora pithya*	On Gymnosperms (*Pinaceae*), rarely on *Salix*	CBS 276.56	MZ233428	MH857634	MH869179	–	Boidin et al. (1991)
		B1013	–	MN475160	MN475827	–	
		CBS 275.56	MZ233427	MH857633	MH869178	–	
*Peniophora polygonia*	*Populus* (*Salicaceae*)	He3668	–	MH669233	MH669237	–	Boidin (1994)
		CBS 404.50	–	MH856684	MH868201	–	Boidin (1994)
*Peniophora proxima*	*Buxus* (*Buxaceae*)	CBS 406.50	–	MH856686	MH868203	–	Boidin (1994)
		CBS 405.50	–	MH856685	MH868202	–	
*Peniophora pseudo-pini*	*Pinus*, *Abies*, *Pseudotsuga*	B1025	–	MN475164	MN475830	–	Gibson (1960)
		DAOM-30124	–	MK588784	MK588824	–	Gibson (1960)
		B1024	–	MN475163	MN475829	–	Gibson (1960)
		B1007	–	MN475162	MN475828	–	Gibson (1960)
*Peniophora pseudonuda*	*Quercus*, *Fagus* (*Fagaceae*)	FCUG 2384	–	GU322866	–	–	Boidin (1994)
		FCUG 2390	–	GU322865	–	–	Boidin (1994)
		FCUG 86	–	GU322867	–	–	Boidin (1994)
*Peniophora pseudoversicolor*	*Quercus* (*Fagaceae*)	CBS 125881	–	MH864303	MH875753	–	Boidin (1994)
		CBS 338.66	–	MH858814	MH870453	–	Boidin (1994)
*Peniophora quercina*	*Betula*, *Castanea*, *Fagus*, *Salix*	CBS 409.50	–	MH856689	MH868206	–	Boidin (1994)
		CBS 408.50	–	MH856688	MH868205	–	Boidin (1994)
		CBS 407.50	–	MH856687	MH868204	–	Boidin (1994)
*Peniophora reidii*	*Quercus* (*Fagaceae*), *Laurus*, *Betula*, *Salix*, *Fagus*, *Eucalyptus*	CBS 397.83	–	MH861616	MH873334	–	Boidin (1994)
*Peniophora rufa*	*Populus tremuloides* (*Salicaceae*)	CBS 351.59	–	MH857891	MH869432	–	Chamuris & Falk (198)
		B1014	–	MN475165	MN475831	–	Chamuris & Falk (198)
*Peniophora rufomarginata*	*Quercus*, *Populus*, *Tilia* and *Arbutrus* (*Ericaceae*)	CBS 282.56	–	MH857640	MH869184	–	Andreasen & Hellenberg (2009)
		CBS 281.56	–	MH857639	MH869183	–	Andreasen & Hellenberg (2009)
*Peniophora septentrionalis*	*Picea*, *Abies* (*Pinaceae*)	CBS 294.58	MZ233429	MH857791	MH869325	–	Andreasen & Hellenberg (2009)
*Peniophora simulans*	*Fagus*	CBS 875.84	–	MH861850	MH873538	–	Reid (1969)
		CBS 874.84	–	MH861849	MH873537	–	Reid (1969)
*Peniophora subsalmonea*	*Mimosaceae*	CBS 697.91	–	MH862303	–	–	Andreasen & Hellenberg (2009)
		CBS 696.91	–	MH862302	–	–	Andreasen & Hellenberg (2009)
*Peniophora taiwanensis*	Angisosperms	Wu 9206 28	–	MK588793	MK588833	–	Andreasen & Hellenberg (2009)
		Wu 9209 14	–	MK588794	MK588834	–	Andreasen & Hellenberg (2009)
*Peniophora tamaricicola*	*Tamarix* (*Tamaricaceae*)	CBS 439.62	–	MH858204	MH869803	–	Gilbertson (1975)
		CBS 441.62	–	MH858205	MH869804	–	Gilbertson (1975)
		CBS 438.62	–	MH858203	MH869802	–	Gilbertson (1975)
*Peniophora versicolor*	*Salix* (*Salicaceae*), *Acer* (*Sapindaceae*), *Ostrya* (*Betulaceae*), *Celtis* (*Cannabaceae*), *Robinia* (*Fabaceae*) and *Ceratonia* (*Fabaceae*)	CBS 358.61	–	MH858082	MH869651	–	Boidin (1994)
*Peniophora violaceolivida*	*Salicaceae,* rarely on “Gymnosperms”	CBS 348.52	–	MH857077	MH868613	–	Andreasen & Hellenberg (2009)

## Data Availability

The data presented in this study is available at Treebase at http://purl.org/phylo/treebase/phylows/study/TB2:S29025.

## Supplementary Materials

The following are available online at https://www.mdpi.com/article/10.3390/jof7121043/s1, Figure S1. Maximum likelihood tree showing Russulales clade obtained from RAxML analyses with a concatenated dataset of 2-loci (LSU and ITS). Table S1. Species used in the *Russulales* analyses and their GenBank accession numbers. Table S2. Heatmap showing the genetic similarities within *Entomocorticium* species.

## References

[1] Aanen D.K., Eggleton P. (2005). Fungus-growing termites originated in African rain forest. Curr. Biol..

[2] Biedermann P.H., Vega F.E. (2020). Ecology and evolution of insect–fungus mutualisms. Annu. Rev. Entomol..

[3] Six D.L., Poulsen M., Hansen A.K., Wingfield M.J., Roux J., Eggleton P., Slippers B., Paine T.D. (2011). Anthropogenic effects on insect-microbial symbioses in forest and savanna ecosystems. Symbiosis.

[4] Mueller U.G., Gerardo N.M., Aanen D.K., Six D.L., Schultz T.R. (2005). The Evolution of Agriculture in Insects. Ann. Rev. Ecol. Evol. Syst..

[5] Hulcr J., Stelinski L.L. (2017). The ambrosia symbiosis: From evolutionary ecology to practical management. Ann. Rev. Entomol..

[6] Franke-Grosman H. (1956). Hautdrüsen als träger der pilzsymbiose bei ambrosiakäfern. Zoomorphology.

[7] Batra L.R. (1963). Ecology of ambrosia fungi and their dissemination by beetles. Trans. Kans. Acad. Sci..

[8] Six D.L. (2003). Bark beetle-fungus symbioses. Insect Symbiosis.

[9] Gomez D.F., Sathyapala S., Hulcr J. (2020). Towards Sustainable Forest Management in Central America: Review of Southern Pine Beetle (*Dendroctonus* frontalis Zimmermann) Outbreaks, Their Causes, and Solutions. Forests.

[10] Thatcher R.C., Searcy J.L., Coster J.E., Hertel G.D. (1980). The Southern Pine Beetle. USDA, Expanded Southern Pine Beetle Research and Application Program, Forest Service, Science and Education Administration, Pineville, LA. Technical. Bull..

[11] Coulson R.N., Klepzig K.D. (2011). Southern Pine Beetle II. General Technical Report.

[12] Hofstetter R.W., Cronin J.T., Klepzig K.D. (2006). Antagonisms, mutualisms, and commensalisms affect outbreak dynamics of the southern pine beetle. Oecologia.

[13] Six D.L. (2012). Ecological and Evolutionary Determinants of Bark Beetle–Fungus Symbioses. Insects.

[14] Whitney H.S., Bandoni R.J., Oberwinkler F. (1987). *Entomocorticium dendroctoni* gen. et sp. nov. (Basidiomycotina), a possible nutritional symbiote of the mountain pine beetle in lodgepole pine in British Columbia. Can. J. Bot..

[15] Harrington T.C., Batzer J.C., McNew D.L. (2021). Corticioid basidiomycetes associated with bark beetles, including seven new Entomocorticium species from North America and *Cylindrobasidium ipidophilum*, comb. nov. Antonie Van Leeuwenhoek.

[16] Hsiau P.T.W., Harrington T.C. (2003). Phylogenetics and adapations of basidiomycetous fungi fed upon by bark beetles (Coleoptera: Scolytidae). Symbiosis.

[17] Victor J., Zuniga G. (2016). Phylogeny of *Dendroctonus* bark beetles (Coleoptera: Curculionidae: Scolytinae) inferred from morphological and molecular data. Syst. Entomol..

[18] Godefroid M., Meseguer A.S., Sauné L., Genson G., Streito J.C., Rossi J.P., Riverón A.Z., Mayer F., Cruaud A., Rasplus J.Y. (2019). Restriction-site associated DNA markers provide new insights into the evolutionary history of the bark beetle genus *Dendroctonus*. Mol. Phylogenet. Evol..

[19] Whitney H.S., Farris F.H. (1970). Maxillary Mycangium in the Mountain Pine Beetle. Science.

[20] Six D.L., Klepzig K.D. (2004). *Dendroctonus* bark beetles as model systems for studies on symbiosis. Symbiosis.

[21] Barras S.J., Perry T.J. (1972). Fungal symbionts in the prothoracic mycangium of *Dendroctonus frontalis* (Coleoptera: Scolytidae). Xeitschrift Fur Angewande Entomol..

[22] Yuceer C., Hsu C.Y., Erbilgin N., Klepzig K.D. (2011). Ultrastructure of the mycangium of the southern pine beetle, *Dendroctonus frontalis* (Coleoptera: Curculionidae, Scolytinae): Complex morphology for complex interactions. Acta Zool..

[23] Furniss M.M., Woo J.Y., Deyrup M.A., Atkinson T.H. (1987). Prothoracic mycangium on pine-infesting *Pityoborus* spp. (Coleoptera: Scolytidae). Ann. Entomol. Soc. Am..

[24] Gouger R.J., Yearian W.C., Wilkinson R.C. (1975). Feeding and reproductive behavior of *Ips avulsus*. Fla. Entomol..

[25] Bracewell R.R., Six D.L. (2014). Broadscale specificity in a bark beetle-fungal symbiosis: A spatio-temporal analysis of the mycangial fungi of the western pine beetle. Microb. Ecol..

[26] Wood S.L. (1982). The bark and Ambrosia Beetles of North and Central America (Coleoptera: Scolytidae), a Taxonomic Monograph Volume 6.

[27] Armendáriz-Toledano F., Niño A., Sullivan B.T., Kirkendall L.R., Zúñiga G. (2015). A new species of bark beetle, Dendroctonus mesoamericanus sp. nov. (Curculionidae: Scolytinae), in southern Mexico and Central America. Ann. Entomol. Soc. Am..

[28] Armendáriz-Toledano F., Zúñiga G. (2017). Illustrated key to species of genus Dendroctonus (Coleoptera: Curculionidae) occurring in Mexico and Central America. J. Insect Sci..

[29] Chen J.J., Cui B.K., Dai Y.C. (2016). Global diversity and molecular systematics of *Wrightoporia* s.l. (Russulales, Basidiomycota). Persoonia.

[30] Leal-Dutra C.A., Neves M.A., Griffith G.W., Reck M.A., Clasen L.A., Dentinger B.T.M. (2018). Reclassification of *Parapterulicium* Corner (Pterulaceae, Agaricales), contributions to Lachnocladiaceae and Peniophoraceae (Russulales) and introduction of *Baltazaria* gen. nov. MycoKeys.

[31] Vilgalys R., Hester M. (1990). Rapid genetic identification and mapping of enzymatically amplified ribosomal DNA from several *Cryptococcus* species. J. Bacteriol..

[32] White T.J., Bruns T.D., Lee S.B., Taylor J.W., Innis M.A., Gelfand D.H., Sninsky J.J. (1990). Amplification and direct sequencing of fungal ribosomal RNA genes for phylogenetics. PCR Protocols: A Guide to Methods and Applications.

[33] Gardes M., Bruns T.D. (1993). ITS primers with enhanced specificity for basidiomycetes-application to the identification of mycorrhizae and rusts. Mol. Ecol..

[34] Kearse M., Moir R., Wilson A., Stones-Havas S., Cheung M., Sturrock S., Buxton S., Cooper A., Markowitz S., Duran C. (2012). Geneious basic: An integrated and extendable desktop software platform for the organization and analysis of sequence data. Bioinformatics.

[35] Katoh K., Rozewicki J., Yamada K.D. (2019). MAFFT online service: Multiple sequence alignment, interactive sequence choice and visualization. Brief. Bioinform..

[36] Stamatakis A. (2006). RAxML-VI-HPC: Maximum likelihood-based phylogenetic analyses with thousands of taxa and mixed models. Bioinform.

[37] Maddison W.P., Maddison D.R. (2018). Mesquite: A Modular System for Evolutionary Analysis. http://www.mesquiteproject.org.

[38] Huson H.H., Scornavacca C. (2012). Dendroscope 3: An Interactive Tool for Rooted Phylogenetic Trees and Networks. Syst. Biol..

[39] Nikoh N., Fukatsu T. (2000). Interkingdom host jumping underground: Phylogenetic analysis of entomoparasitic fungi of the genus *Cordyceps*. Mol. Biol. Evol..

[40] Chaverri P., Samuels G.J. (2013). Evolution of habitat preference and nutrition mode in a cosmopolitan fungal genus with the evidence of interkingdom host jumps and major shifts in ecology. Evolution.

[41] Skelton J., Jusino M.A., Li Y., Bateman C., Thai P.H., Wu C., Lindner D.L., Hulcr J. (2018). Detecting Symbioses in Complex Communities: The Fungal Symbionts of Bark and Ambrosia Beetles Within Asian Pines. Microb. Ecol..

[42] Spatafora J.W., Sung G.H., Sung J.M., Hywel-Jones N., White Jr J.F. (2007). Phylogenetic evidence for an animal pathogen origin of ergot and the grass endophytes. Mol. Ecol..

[43] O’Donnell K., Sink S., Libeskind-Hadas R., Hulcr J., Kasson M.T., Ploetz R.C., Konkol J.L., Ploetz J.N., Carrillo D., Campbell A. (2015). Discordant phylogenies suggest repeated host shifts in the *Fusarium*-*Euwallaceae* ambrosia beetle mutualism. Fungal Genet. Biol..

[44] Araújo J.P.M., Hughes D.P. (2019). Zombie-ant fungi emerged from non- manipulating, beetle-infecting ancestors. Curr. Biol..

[45] Araújo J.P.M., Moriguchi M.G., Uchyiama S., Kinjo N., Matsuura Y.M. (2021). *Ophiocordyceps salganeicola*, a parasite of social cockroaches in Japan and insights into the evolution of other closely-related *Blattodea*-associated lineages. IMA Fungus.

[46] Skelton J., Johnson A.J., Jusino M.A., Bateman C.C., Li Y., Hulcr J. (2019). A selective fungal transport organ (mycangium) maintains coarse phylogenetic congruence between fungus-farming ambrosia beetles and their symbionts. Proc. R. Soc. B.

[47] Lee S., Kim J.-J., Breuil C. (2006). Diversity of fungi associated with the mountain pine beetle, *Dendroctonus ponderosae*, and infected lodgepole pines in British Columbia. Fungal Divers..

[48] Roe A.R., James P.M.A., Rice A.V., Cooke J.E.K., Sperling F.H. (2011). Spatial community structure of mountain pine beetle fungal symbionts across a latitudinal gradient. Microb. Ecol..

[49] Six D.L., Bentz B.J. (2007). Temperature determines symbiont abundance in a multipartner bark beetle-fungus ectosymbiosis. Microb. Ecol..

[50] Bracewell R.R., Six D.L. (2019). Experimental evidence of bark beetle adaptation to a fungal symbiont. Ecol. Evol..

[51] Bracewell R.R., Vanderpool D., Good J., Six D.L. (2018). Cascading speciation among mutualists and antagonists in a tree-beetle-fungal interaction. R. Soc. Proc. B.

[52] Six D.L., Elser J.J. (2019). Extreme ecological stoichiometry of a bark beetle-fungus mutualism. Ecol. Entomol..

[53] Six D.L. (2020). A major symbiont shift supports a major niche shift in a clade of tree-killing bark beetles. Ecol. Entomol..

[54] Van de Peppel L.J., Aanen D.K., Biedermann P.H. (2018). Low intraspecific genetic diversity indicates asexuality and vertical transmission in the fungal cultivars of ambrosia beetles. Fungal Ecol..

[55] Matsuura Y., Moriyama M., Łukasik P., Vanderpool D., Tanahashi M., Meng X.Y., McCutcheon J.P., Fukatsu T. (2018). Recurrent symbiont recruitment from fungal parasites in cicadas. Proc. Nat. Acad. Sci. USA.

[56] Boidin J., Languetin P., Gilles G. (1991). Les Peniophoraceae de la zone intertropicale (Basidiomycetes, Aphyllophorales). Bull. Soc. Mycol. Fr..

[57] Andreasen M., Hallenberg N. (2009). A taxonomic survey of the Peniophoraceae. Synop. Fungorum.

[58] Muller H.J. (1964). The relation of recombination to mutational advance. Mutat. Res..

[59] Bidochka M.J., De Koning J. (2001). Are teleomorphs really necessary? Modelling the potential effects of Muller’s Ratchet on deuteromycetous entomopathogenic fungi. Mycol. Res..

[60] Hofstetter R.W., Gandhi K.J.K. (2021). Bark Beetle Management, Ecology, and Climate Change.

[61] Six D.L., Klepzig K. (2021). Context dependency in bark beetle-fungus symbioses revisited: Assessing potential shifts in interaction outcomes against varied genetic, ecological, and evolutionary backgrounds. Front. Microbiol..

[62] Farjon A. (1996). Biodiversity of *Pinus* (Pinaceae) in Mexico: Speciation and palaeo-endemism. Bot. J. Linn. Soc..

