# Supplementary material for: Diversity and Evolution of Entomocorticium (Russulales, Peniophoraceae), a Genus of Bark Beetle Mutualists Derived from Free-Living, Wood Rotting Peniophora
Source: J Fungi (Basel). 2021 Dec 6;7(12):1043. doi: 10.3390/jof7121043 (PMC8706356; doi:10.3390/jof7121043)
Supplement: Supplementary file 1 [file jof-07-01043-s001.zip › Supplementary Figure S1.pdf]

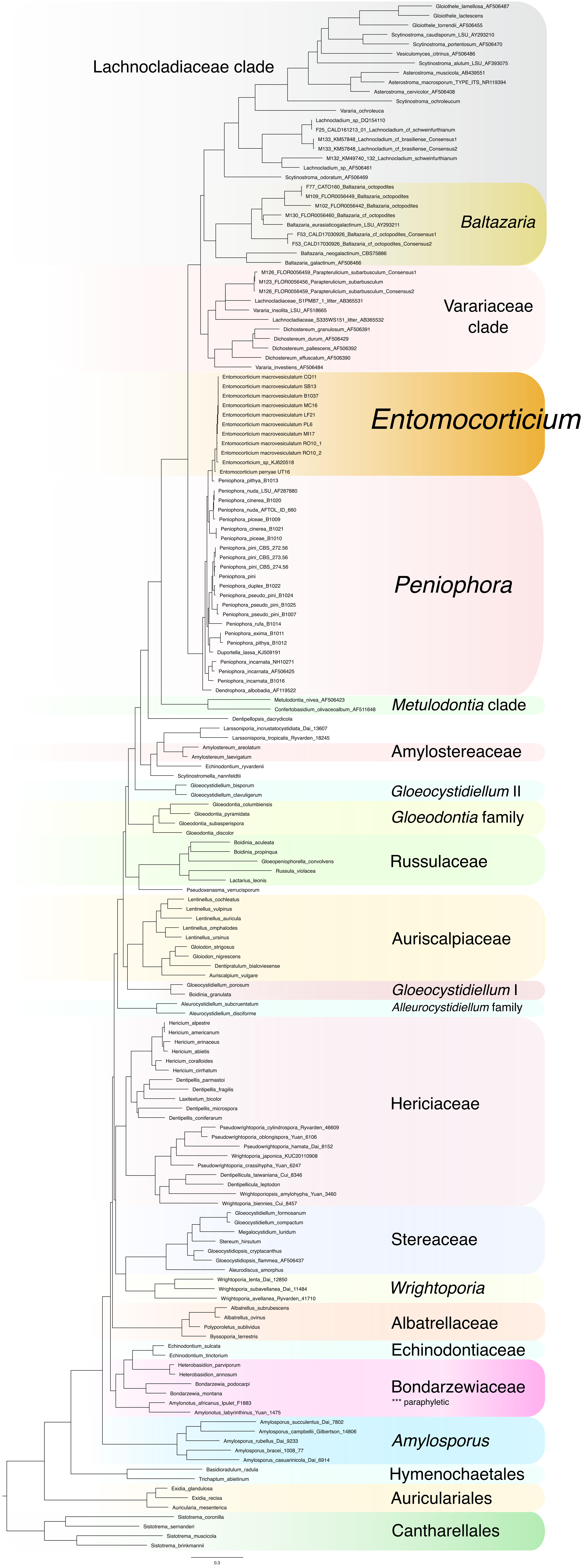

Gloiothele_lamellosa_AF506487
Gloiothele_lactescens
Gloiothele_torrendii_AF506455
Scytinostroma_caudisporum_LSU_AY293210
Scytinostroma_portentosum_AF506470
Vesiculomyces_citrinus_AF506486
Scytinostroma_alutum_LSU_AF393075
Asterostroma_muscicola_AB439551
Asterostroma_macrosporum_TYPE_ITS_NR119394
Asterostroma_cervicolor_AF506408
Scytinostroma_ochroleucum
Vararia_ochroleuca
Lachnocladium_sp_DQ154110
F25_CALD161213_01_Lachnocladium_cf_schweinfurthianum
M133_KM57848_Lachnocladium_cf_brasiliense_Consensus1
M133_KM57848_Lachnocladium_cf_brasiliense_Consensus2
M132_KM49740_132_Lachnocladium_schweinfurthianum
Lachnocladium_sp_AF506461
Scytinostroma_odoratum_AF506469
Lachnocladiaceae clade
F77_CATO160_Baltazaria_octopodites
M109_FLOR0056449_Baltazaria_octopodites
M102_FLOR0056442_Baltazaria_octopodites
M130_FLOR0056460_Baltazaria_cf_octopodites
Baltazaria_eurasiaticogalactinum_LSU_AY293211
F53_CALD17030926_Baltazaria_cf_octopodites_Consensus1
F53_CALD17030926_Baltazaria_cf_octopodites_Consensus2
Baltazaria_neogalactinum_CBS75886
Baltazaria_galactinum_AF506466
Baltazaria
M126_FLOR0056459_Parapterulicium_subarbusculum_Consensus1
M123_FLOR0056456_Parapterulicium_subarbusculum
M126_FLOR0056459_Parapterulicium_subarbusculum_Consensus2
Lachnocladiaceae_S1PMB7_1_litter_AB365531
Vararia_insolita_LSU_AF518665
Lachnocladiaceae_S335WS151_litter_AB365532
Dichostereum_granulosum_AF506391
Dichostereum_durum_AF506429
Dichostereum_pallescens_AF506392
Dichostereum_effuscatum_AF506390
Vararia_investiens_AF506484
Varariaceae clade
Entomocorticium macrovesiculatum CQ11
Entomocorticium macrovesiculatum SB13
Entomocorticium macrovesiculatum B1037
Entomocorticium macrovesiculatum MC16
Entomocorticium macrovesiculatum LF21
Entomocorticium macrovesiculatum PL6
Entomocorticium macrovesiculatum MI17
Entomocorticium macrovesiculatum RO10_1
Entomocorticium macrovesiculatum RO10_2
Entomocorticium_sp_KJ620518
Entomocorticium perryae UT16
Entomocorticium
Peniophora_pithya_B1013
Peniophora_nuda_LSU_AF287880
Peniophora_cinerea_B1020
Peniophora_nuda_AFTOL_ID_660
Peniophora_piceae_B1009
Peniophora_cinerea_B1021
Peniophora_piceae_B1010
Peniophora_pini_CBS_272.56
Peniophora_pini_CBS_273.56
Peniophora_pini_CBS_274.56
Peniophora_pini
Peniophora_duplex_B1022
Peniophora_pseudo_pini_B1024
Peniophora_pseudo_pini_B1025
Peniophora_pseudo_pini_B1007
Peniophora_rufa_B1014
Peniophora_exima_B1011
Peniophora_pithya_B1012
Duportella_lassa_KJ509191
Peniophora_incarnata_NH10271
Peniophora_incarnata_AF506425
Peniophora_incarnata_B1016
Dendrophora_albobadia_AF119522
Peniophora
Metulodontia_nivea_AF506423
Confertobasidium_olivaceoalbum_AF511648
Metulodontia clade
Dentipellopsis_dacrydicola
Larssoniporia_incrustatocystidiata_Dai_13607
Larssonisporia_tropicalis_Ryvarden_18245
Amylosteum_areolatum
Amylostereum_laevigatum
Echinodontium_ryvardenii
Scytinostromella_nannfeldtii
Amylostereaceae
Gloeocystidiellum_bisporum
Gloeocystidiellum_clavuligerum
Gloeocystidiellum II
Gloeodontia_columbiensis
Gloeodontia_pyramidata
Gloeodontia_subasperispora
Gloeodontia_discolor
Gloeodontia family
Boidinia_aculeata
Boidinia_propinqua
Gloeopeniophorella_convolvens
Russula_violacea
Lactarius_leonis
Russulaceae
Pseudoxenasma_verrucisporum
Lentinellus_cochleatus
Lentinellus_vulpinus
Lentinellus_auricula
Lentinellus_omphalodes
Lentinellus_ursinus
Gloiodon_strigosus
Gloiodon_nigrescens
Dentipratulum_bialoviesense
Auriscalpium_vulgare
Auriscalpiaceae
Gloeocystidiellum_porosum
Boidinia_granulata
Gloeocystidiellum I
Aleurocystidiellum_subcruentatum
Aleurocystidiellum_disciforme
Alleurocystidiellum family
Hericium_alpestre
Hericium_americanum
Hericium_erinaceus
Hericium_abietis
Hericium_coralloides
Hericium_cirrhatum
Dentipellis_parmastoi
Dentipellis_fragilis
Laxitextum_bicolor
Dentipellis_microspora
Dentipellis_coniferarum
Pseudowrightoporia_cylindrospora_Ryvarden_46609
Pseudowrightoporia_oblongispora_Yuan_6106
Pseudowrightoporia_hamata_Dai_8152
Wrightoporia_japonica_KUC20110908
Pseudowrightoporia_crassihypha_Yuan_6247
Dentipellicula_taiwaniana_Cui_8346
Dentipellicula_leptodon
Wrightoporiopsis_amylohypha_Yuan_3460
Wrightoporia_biennies_Cui_8457
Hericiaceae
Gloeocystidiellum_formosanum
Gloeocystidiellum_compactum
Megalocystidium_luridum
Stereum_hirsutum
Gloeocystidiopsis_cryptacanthus
Gloeocystidiopsis_flammea_AF506437
Aleurodiscus_amorphus
Stereaceae
Wrightoporia_lenta_Dai_12850
Wrightoporia_subavellanea_Dai_11484
Wrightoporia_avellanea_Ryvarden_41710
Wrightoporia
Albatrellus_subrubescens
Albatrellus_ovinus
Polyporoletus_sublividus
Byssoporia_terrestris
Albatrellaceae
Echinodontium_sulcata
Echinodontium_tinctorium
Echinodontiaceae
Heterobasidion_parviporum
Heterobasidion_annosum
Bondarzewia_podocarpi
Bondarzewia_montana
Amylonotus_africanus_Ipulet_F1883
Amylonotus_labyrinthinus_Yuan_1475
Bondarzewiaceae
*** paraphyletic
Amylosporus_succulentus_Dai_7802
Amylosporus_campbellii_Gilbertson_14806
Amylosporus_rubellus_Dai_9233
Amylosporus_bracei_1008_77
Amylosporus_casuarinicola_Dai_6914
Amylosporus
Basidioradulum_radula
Trichaptum_abietinum
Hymenochaetales
Exidia_glandulosa
Exidia_recisa
Auricularia_mesenterica
Auriculariales
Sistotrema_coronilla
Sistotrema_sernanderi
Sistotrema_muscicola
Sistotrema_brinkmannii
Cantharellales
0.3
